# Catastrophic Health Care Expenditure among Older People with Chronic Diseases in 15 European Countries

**DOI:** 10.1371/journal.pone.0157765

**Published:** 2016-07-05

**Authors:** Jelena Arsenijevic, Milena Pavlova, Bernd Rechel, Wim Groot

**Affiliations:** 1 Department of Health Services Research, CAPHRI, Maastricht University Medical Center, Faculty of Health, Medicine and Life Sciences, Maastricht University, Maastricht, The Netherlands; 2 European Observatory on Health Systems and Policies, London School of Hygiene and Tropical Medicine, London, United Kingdom; 3 Top Institute Evidence-Based Education Research (TIER), Maastricht University, Maastricht, The Netherlands; University of Perugia, ITALY

## Abstract

**Introduction:**

It is well-known that the prevalence of chronic diseases is high among older people, especially those who are poor. Moreover, chronic diseases can result in catastrophic health expenditure. The relationship between chronic diseases and their financial burden on households is thus double-sided, as financial difficulties can give rise to, and result from, chronic diseases. Our aim was to examine the levels of catastrophic health expenditure imposed by private out-of-pocket payments among older people diagnosed with diabetes mellitus, cardiovascular diseases and cancer in 15 European countries.

**Methods:**

The SHARE dataset for individuals aged 50+ and their households, collected in 2010–2012 was used. The total number of participants included in this study was N = 51,661. The sample consisted of 43.8% male and 56.2% female participants. The average age was 67 years. We applied an instrumental variable approach for binary instrumented variables known as a treatment-effect model.

**Results:**

We found that being diagnosed with diabetes mellitus and cardiovascular diseases was associated with catastrophic health expenditure among older people even in comparatively wealthy countries with developed risk-pooling mechanisms. When compared to the Netherlands (the country with the lowest share of out-of-pocket payments as a percentage of total health expenditure in our study), older people diagnosed with diabetes mellitus in Portugal, Poland, Denmark, Italy, Switzerland, Belgium, the Czech Republic and Hungary were more likely to experience catastrophic health expenditure. Similar results were observed for diagnosed cardiovascular diseases. In contrast, cancer was not associated with catastrophic health expenditure.

**Discussion:**

Our study shows that older people with diagnosed chronic diseases face catastrophic health expenditure even in some of the wealthiest countries in Europe. The effect differs across chronic diseases and countries. This may be due to different socio-economic contexts, but also due to the specific characteristics of the different health systems. In view of the ageing of European populations, it will be crucial to strengthen the mechanisms for financial protection for older people with chronic diseases.

## Introduction

Chronic diseases are a leading cause of death in high-income countries and a growing problem in other parts of the world [1, 2]. The prevalence of chronic diseases is higher among older people, especially those who are poor [3, 4]. Chronic diseases can result in catastrophic health care expenditure and impose a substantial financial burden. At the same time, catastrophic spending can lead to chronic disease [3, 5]. Households that experience catastrophic health expenditure may face poverty and need to cut on other types of expenditure (food, education etc.) or to use coping mechanisms such as savings or borrowing money [6]. In this way, members of those households become more vulnerable to additional chronic diseases or related comorbidities [7]. This suggests that the relation between chronic diseases and is catastrophic health expenditure imposed by them complex and can act in both directions, with chronic diseases either resulting from, or leading to, financial difficulties.

Catastrophic health expenditures refer to the case when out-of-pocket payments exceed a certain threshold share of either total or non-food expenditure of households [7]. The choice of threshold is somewhat arbitrary, but commonly used thresholds are 10–25% of total consumption expenditure or 25–40% of non-food expenditure [8]. Previous studies have also used different indicators of wealth such as expenditure, income and consumption. Although, there is also no clear consensus which indicator of wealth is the best, previous studies have shown that expenditure is a more accurate indicator in countries with a large informal economy. In those countries, reported income (money received officially from the current job) is just part of the total income. Usually, there is also a part of the salary that is not registered with the tax system. In countries with a developed tax system, income is more often used as an indicator of wealth. In those countries, there is no or only limited possibility to have an informal income. In this way, reported income represents households’ ability to pay for health care without using coping mechanisms such as borrowing or selling assets [9]. Another approach to assess the financial burden resulting from out-of-pocket payments for health would be to measure the incidence of households that fall below the national poverty line as a result, known as the impoverishing effect of out-of-pocket expenditure [10]. The existing literature suggests that the incidence of catastrophic health expenditure is a more appropriate measure of the financial burden of out-of-pocket health expenditure in developed countries (European countries or the United States), while impoverishment is used to assess the financial burden in developing countries [7].

In this study, we aim to assess the level of catastrophic health expenditure provoked by private out-of-pocket payments among older people diagnosed with chronic diseases in 15 European countries (Austria, Belgium, Czech Republic, Denmark, France, Germany, Hungary, Italy, Netherlands, Poland, Portugal, Spain, Slovenia, Sweden and Switzerland). We use catastrophic health expenditure as our approach, since in most European Union (EU) countries existing protection mechanisms prevent impoverishment. We apply a threshold of 10% of total household expenditure per person and year, which is the most commonly used threshold for catastrophic health expenditure in EU countries [11–13] and income as an indicator of wealth. To account for joint causality between catastrophic health expenditure and chronic diseases, we use an instrumental variable approach.

The catastrophic health expenditure of chronic diseases is a particular problem in health systems that rely to a large extent on (formal or informal) patient charges or co-payments [5, 10, 14–17]. Although health systems in Europe display great diversity in how they are financed, most require obligatory co-payments for basic services related to chronic diseases [18]. However, there are major differences not only with regard to the presence, but also the scope and magnitude of private co-payments [19, 20]. Table 1 illustrates the scope and magnitude of private co-payments, as well as the share of private out-of-pocket payments as a percentage of total health expenditure and of GDP in the 15 European countries considered in this study. The table also shows where older people and those with chronic or severe illness are exempted from official co-payments.

**Table 1:** Macro indicators on health expenditure in 15 European countries.

	Total health expenditure as a percentage of GDP ^a^	Government expenditure on health as a percentage of GDP ^a^	Private health expenditure as a percentage of total health expenditure ^a^	Out-of-pocket payments as a percentage of total health expenditure ^a^	Out-of-pocket payments as a percentage of private expenditure ^a^	Scope of formal patient co-payments ^b^^,^^c^	Exemption or reduction of co-payments for elderly ^b^	Exemption or reduction of co-payments for chronic or severe illness ^b^	Presence of informal patient payments ^b^
**Austria**	11%	9%	24%	15%	62%	Broad scope	Partly present	Present	Some
**Belgium**	11%	8%	24%	20%	82%	Broad scope	Partly present	Present	No
**Czech Republic**	8%	6%	15%	14%	93%	Broad scope	Partly present	Present	Some
**Denmark**	11%	10%	14%	13%	87%	Narrow scope	No co-payments	No co-payments	No
**France**	12%	9%	23%	7%	32%	Broad scope	Partly present	Present	Some
**Germany**	11%	9%	24%	12%	51%	Broad scope	Not present	Present	No
**Hungary**	8%	5%	36%	27%	74%	Narrow scope	No co-payments	No co-payments	Widespread
**Italy**	9%	7%	22%	20%	93%	Broad scope	Partly present	Present	Some
**Netherlands**	12%	10%	13%	6%	42%	Broad scope	Not present	Present	No
**Poland**	7%	5%	30%	23%	76%	Narrow scope	No co-payments	No co-payments	Widespread
**Portugal**	9%	6%	37%	32%	85%	Broad scope	Partly present	Present	No
**Spain**	10%	7%	26%	20%	77%	Narrow scope	No co-payments	No co-payments	No
**Slovenia**	9%	6%	27%	12%	45%	Broad scope	Partly present	Present	No
**Sweden**	10%	8%	18%	16%	88%	Broad scope	Partly present	Present	No
**Switzerland**	11%	7%	38%	28%	73%	Broad scope	-	-	no

^a^ Source WHO,2012, We use data for 2012, since this is the most recent year in which data are available for all countries included in this study

^b^ Tambor et al, 2013,

^c^ Narrow scope = no obligatory co-payments for services in the basic package (GP services, specialist services and inpatient care except for dentist); Broad scope = obligatory co-payments for these services are present.

Total health expenditure as a share of GDP varied across the 15 European countries from 7% to 12% for year 2012 (Table 1). The share of private out-of-pocket payments as a percentage of total health expenditure varied from 6% in the Netherlands to 32% in Portugal. Only Denmark, Hungary, Poland and Spain have no official co-payments for basic services (such as visits to general practitioners -GPs). However, Denmark and Spain are the only two countries where the scope of official co-payments is limited and no informal payments exist. In contrast, informal payments are reported to be widespread in Hungary and Poland, thus apparently making up for the limited scope of official co-payments [21].

Based on the contextual information presented in Table 1, we expect that the incidence of catastrophic health expenditure is lower in countries without extensive official co-payments and without informal payments. Previous studies suggest that the incidence of catastrophic health expenditure is higher in those countries where the share of private out-of-pocket payments in total health care expenditure is higher than 20% (such as in Hungary, Poland, Portugal and Switzerland), and lower in countries where the share of out-of-pocket payments is lower (such as in the Netherlands) [21, 22]. We also expect that in those countries where government spending on health care is lower than 10% of GDP, the incidence of catastrophic health expenditure would be higher (such as in Hungary, the Czech Republic, Poland, Portugal and Slovenia) [12]. Exemptions for older people and those with chronic illnesses are expected to be associated with a lower incidence of catastrophic health expenditure. Our aim is to examine the levels of catastrophic health expenditure provoked by private out-of-pocket payments among older people diagnosed with diabetes mellitus, cardiovascular diseases and cancer in 15 European countries. In this way, we also investigated the above expectations.

## Methods

In order to explore the joint causality between chronic diseases and catastrophic health expenditure among older adults in Europe, we used the SHARE dataset for individuals aged 50+ and their households, collected in 2010–2012 (wave 4). In this study we use SHARE data for 15 different European countries. SHARE is an enterprise by researchers for researchers and data are collected by different researchers in 15 EU countries. The data collection is funded by European Commission through the 5th, 6th and 7th framework program. Until July 2011, SHARE has been reviewed and approved by the Ethics Committee of the University of Mannheim. Since then, the Ethics Council of the Max-Planck-Society for the Advancement of Science (MPG) is responsible for ethical reviews and the approval of the study. Those information are provided on web page:http://www.share-project.org/. Wave 4 of the SHARE dataset provides data for individuals aged 50+ and their households in 2010–2012 in 15 European countries (Austria, Belgium, Czech Republic, Denmark, France, Germany, Hungary, Italy, Netherlands, Poland, Portugal, Spain, Slovenia, Sweden and Switzerland). The SHARE data are collected through computer-assisted personal interview techniques that consist of several modules: modules for all household members and individual modules for eligible household members (eligible individuals are persons born in 1960 or earlier, having their regular domicile in the respective country, including that of their current partners/spouses, independent of age) for different topics. The SHARE dataset also includes modules collected through paper and pencil questionnaires, such as for country-specific data related to out-of-pocket payments for health care and generated modules (data regarding social networks and imputation data). These data are collected by the interviewer directly or the questionnaires are sent by post. The sample size per country included is between 1572 (in Germany) and 6118 (in the Czech Republic) respondents. Detailed information about the number of participants in each country is presented in Table A in S1 file.

As the SHARE data represent a cross-national survey that contains detailed information about mental and physical health, housing and expenditure, missing data are unavoidable. Since wave 2, imputation techniques have been used to overcome the problem related to missing data [23]. The imputed data include five different versions for each imputed variable. The imputation procedure in SHARE wave 4 takes in account household composition and other relevant socio-demographic characteristics in calculating the imputed values. Imputation data also provide some aggregate variables such as total household expenditure and total household income. Those variables have been widely used in previous research. Detailed information about the number of missing data for each imputed variable per country can be found in the technical supplements of the SHARE guidelines [23]. Also, more in-depth information about the data collection procedures is available on the SHARE website [23, 24].

For the purpose of this study, we used data from all available modules for eligible individuals.

To address the bias resulting from the joint causality, we applied an instrumental variable approach for binary instrumented variables known as the treatment-effect model. We included in the analysis the three most common chronic diseases, namely diabetes mellitus, cardiovascular diseases and cancer [25–27]. Applying the treatment-effect model for each of the three diseases allows us to estimate the probability of catastrophic health expenditure for people diagnosed with one of the chronic diseases when other factors are controlled for. We first describe the variables that we used in the analysis and then describe the analysis itself.

### Outcome variable

To assess the catastrophic effects of out-of-pocket payments on household budgets, we used the available data regarding these payments from the paper-pencil questionnaire, as well as data related to household income available in the computer-assisted personal interview module. As we mentioned above, catastrophic health expenditure occurs when health spending exceeds a certain threshold within a specified period of time [7]. However, there is no consensus regarding the threshold that should be applied. Recent studies suggest that the threshold can vary from 5% to 40% (11–13). In this study, we applied a threshold of 10% of total household income per year, which is the threshold most commonly used in EU countries [11–13]. To calculate the catastrophic effects of out-of-pocket payments, we first calculated the total amount of out-of-pocket payments per household for each eligible individual per year by summing up out-of-pocket payments for inpatient care, outpatient care, prescribed drugs, care in nursing homes, day care and home care services. Then we divided the total health expenditure by total household income. In case data for out-of-pocket patient payments were missing, we were not able to use imputation data since in Wave 4 there was no imputation for out-of-pocket health care expenditure [23]. However, we were able to use the imputed data for missing income information. We have decided to use available imputed data in order to avoid missing cases for catastrophic health care expenditure in case the respondent reported out-of-pocket patient payments but did not provide information about income. For the missing income cases, imputation data were used in accordance with SHARE guidelines [24]. Since, SHARE data provide information about household health expenditure and income for all households members, we have expressed catastrophic health care expenditure per households for each eligible person.

**Fig 1:**
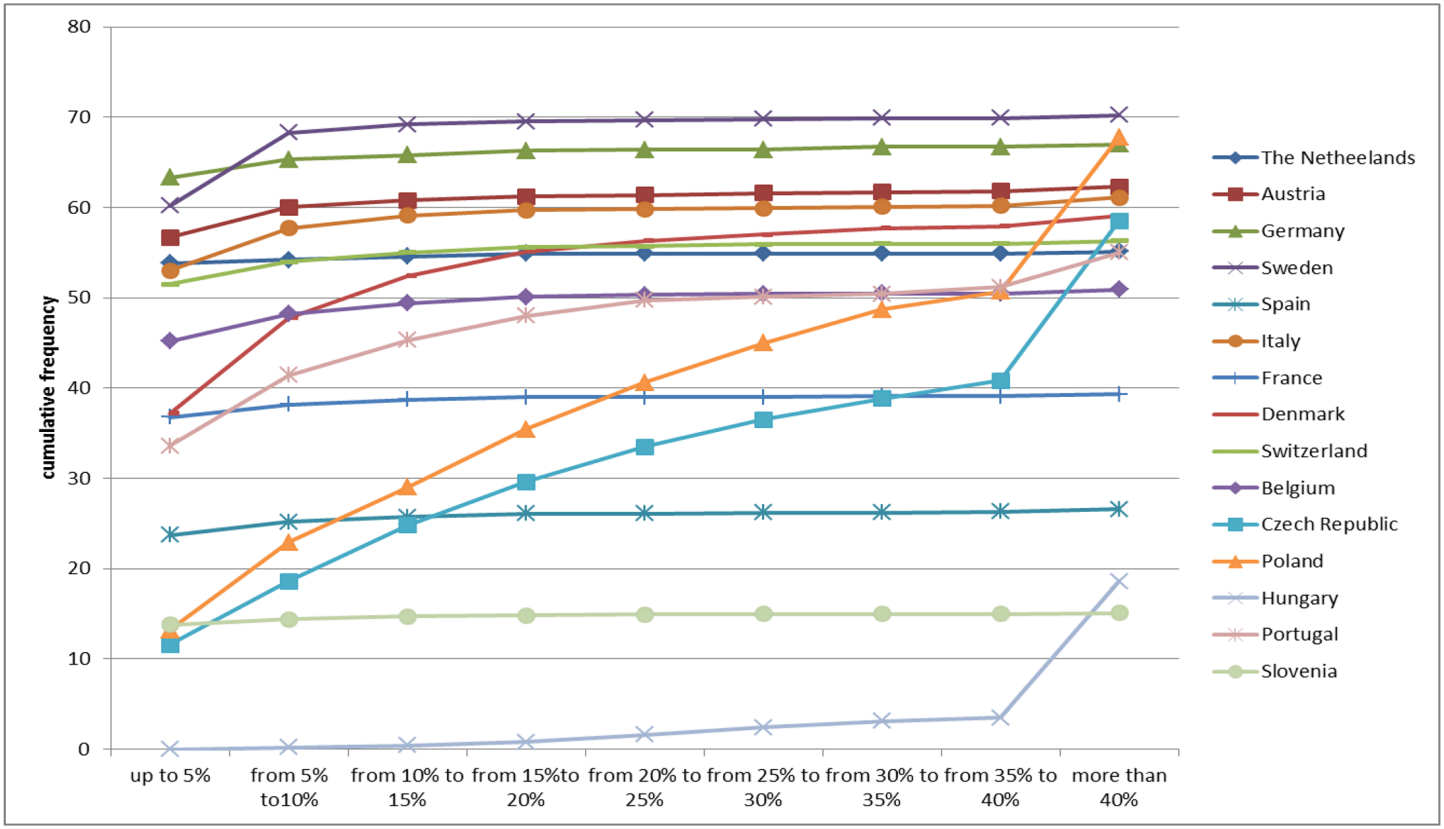
Catastrophic health expenditure in different countries when thresholds varied from 1 = 5%up to 9 = more than 40%. Catastrophic health expenditures refer to the case when out-of-pocket payments exceed a certain threshold share of either total or non-food expenditure of households. Data are presented for thresholds in the ranging from 5% to 40%.

Using the data for out-of-pocket payments and income described above, we created a binary indicator for catastrophic health expenditure. The indicator was coded as 1 if the total amount of out-of-pocket payments per person per year exceeded 10% of total annual income per person. Otherwise, the indicator was coded as 0. This indicator was our outcome variable. Additionally, we created indicators for other catastrophic expenditure thresholds in the ranging from 5% to 40% to check how the incidence of catastrophic expenditure changes with the change of the threshold. These data are presented in Fig 1. We used income as an indicator of wealth, as this is considered adequate in EU countries [10].

### Indicators of chronic diseases

We created binary indicators for three different chronic diseases, using data available from the physical health information in the computer-assisted personal interview module in the SHARE dataset. The binary indicator for diabetes mellitus is coded 1 if the person is diagnosed by diabetes mellitus; otherwise it was coded as 0. We used the same procedure to create the binary indicator regarding diagnosed cancer. For the binary indicator regarding cardiovascular diseases, we coded the indicator as 1, if the respondent was diagnosed with one of the following diseases: heart attack and stroke. Otherwise, the indicator was coded as 0. Since the binary indicators can face the problem of endogeneity, we used a set of instrumental variables [28] to control for issues such as alcohol consumption, smoking, comorbidities, body mass index and country of origin.

### Covariates

As covariates, we used variables on socio-demographic characteristics, specifically gender, age, household size, number of children, years of education and household expenditure percentiles [13, 22, 29–32]. We also created a binary indicator for each country, using the Netherlands as the base country category for comparison, since the share of out-of-pocket payments as a percentage of total health expenditure is lowest in this country (Table 1). Since 15 countries were included in this study, we created 14 different dummy variables: one for each country (for example Austria was coded 1 for households in Austria and 0 for all other observations), except for the Netherlands that was considered as the base group (i.e. the dummy for the Netherlands was not included).

### Treatment-effect model and instrumental variables

Since the relationship between different chronic diseases and catastrophic health expenditure is characterised by a joint causality, we use the treatment–effect model to instrument the indicators of chronic diseases. We assume that indicators of chronic diseases are endogenous. In order to address this endogeneity, we need—exogenous variables instruments. Instruments are related to endogenous predictors (chronic diseases) but are not related to outcome variables [28].

In order to identify good instruments, we first reviewed the existing literature [14, 26, 32–37]. We identified three groups of potential instruments: lifestyle (alcohol consumption, eating habits, smoking, body mass index), comorbidities (high level of cholesterol in blood and hypertension for cardiovascular disease) and country of origin (Asian origin including participants born in Afghanistan, Cambodia, Sri Lanka, China, Hong Kong, India, Indonesia, Japan, Lao People’s Democratic Republic, Macao, Malesia, Pakistan and Singapore and Middle East origin-participants born in Iran, Iraq, Iran, Israel, Lebanon, Syria, Turkey and Egypt) [38, 39].

Good instruments should satisfy two main criteria known as relevance and validity criteria [28]. This means that good instruments would be correlated with the binary indicator of each of the chronic diseases (relevance criteria) but would not be correlated with the error terms in the model of the outcome variable (validity criteria). Based on those criteria, the country of origin is a potentially good instrument in our case, while the suitability of lifestyle can be questioned. For example, lifestyle is often associated with socio-economic status [40]. Since we calculate catastrophic health expenditure using income, this means that life style can be associated also with catastrophic health care expenditure. This is not in accordance with the validity criteria. However, recently published studies show that there is no straightforward association between lifestyle and socio-economic status in older adults [40]. In some cases, people with low-socioeconomic status are more often engaged in an unhealthy lifestyle [41], while in other cases, people with middle and higher socioeconomic status are those who consume alcohol more often or are more likely to be obese [42]. Since the relation between an unhealthy lifestyle and income depends on the type of unhealthy lifestyle, but also on perceptions of health status, we decided to use statistical methods to check the quality, validity and relevance of the potential instruments. To check for the relevance of the instruments, we run the first stage regression as a OLS model using potential instrument variables as predictors of each of the indicators of chronic diseases. We also performed the over-identification or Sargan test in order to examine whether the instruments are correlated with error terms in the second stage regression. Data related to relevance criteria are presented in Table B in S1 File.

In order to check the validity of potential instruments, we first analysed the correlation between potential instruments with the outcome variable (the incidence of catastrophic health expenditure). We considered the instrument to be initially valid if the correlation was lower than ± 0.2 [43, 44]. In order to check the validity of instruments, we also performed the Hausman test [45]. The Hausman test examines whether the endogenous predictor is truly endogenous. In other words, the Hausman test checks the hypothesis if there is any correlation between the error term in the first stage regression and the error term in the second stage regression (H_0_: cov(ε_i_, δi) = 0). If the H_0_ is true both OLS and treatment effect variable estimators are consistent and therefore it is not necessary to use treatment-effect model. If the null hypothesis is rejected, treatment effect model is required [45]. The instruments that are valid and relevant for each of the chronic diseases are included in the treatment-effect model. For diabetes mellitus relevant and valid instruments are alcohol consumption, smoking, body mass index and origin from the Middle East. Relevant criteria for cardiovascular diseases are high level of cholesterol, smoking, body mass index and origin from Asia, and for diagnosed cancer physical activity and smoking. The results regarding the validity tests are presented in Table C in S1 file.

A treatment-effect model consists of a two-stage regression. In the first stage, we regress the binary disease-specific indicators described above (for diabetes mellitus, cardiovascular diseases and cancer) on the instrument variables and covariates. In the second-stage regression, we regress the binary indicators of catastrophic health expenditure on the predicted values of disease-specific indicators (based on the first-stage regression) and the same set of covariates.

If there was no joint causality, we can apply a simple regression analysis, directly using the instrumented variables as the covariates for catastrophic health expenditure. Therefore, we also compare the results from the treatment-effect model with the results of ordinary least square regression (OLS) that is compatible with the second-stage regression of the treatment-effect model.

As we have mentioned before, to calculate the catastrophic health expenditures ratio, we have used imputed values for income. The imputation process brings some risks and potential biases. For example, if part of the aggregated income variable is missing, the variable itself is considered as missing. However, the available information contained in the single items are preserved and used as individual lower bonds. In our sample, income data are imputed for 19222 participants (37.2% of the total sample). Based on this, we have decided to perform sensitivity analyses by omitting the imputed values and by re-running the analyses for all three models (cardio-vascular diseases, diabetes and cancer). We present the results in Table E in S1 file and Table F in S1 file.

Furthermore, we have examined the correlation between the predicted probability of catastrophic health expenditure for people diagnosed with chronic diseases (diabetes mellitus, cardiovascular diseases and cancer) and the macro-indicators presented in Table 1. The predicted probability is estimated by the post estimation test in Stata (treatreg postestimation), indicating the probability of catastrophic health expenditure conditional on one of the three chronic diseases is present. This gives insight in the relation of country–specific macro-indicators and catastrophic health care expenditure imposed by one of three chronic diseases.

## Results

The results of the treatment-effect model are presented in Tables 2, 3 and 4. The association between the macro indicators presented in Table 1 and the predicted probability of catastrophic health expenditure are described in Table 5. Descriptive statistics for all variables that were used in the analyses, as well as validity and relevance tests for instrumental variables are presented in Tables A, B, C and D in S1 File. Specifically in Table D in S1 file, we present descriptive statistics for out-of-pocket payments for different services (including payments for inpatient care, outpatient care, prescribed drags, home care services and nursing home services) for each of the chronic diseases considered in this study in the different countries. These results show the average amount spent on different services for people diagnosed with diabetes mellitus, stroke, high blood pressure, high blood cholesterol, heart attack and cancer. The results from sensitivity analyses are presented in the supplementary materials. Results for treatment–effect model without imputed data are presented in Table E and F in S1 file.

**Table 2:** Results of the treatment-effect model (two-steps); dependent variable: catastrophic effects of health expenditure.

*Second-stage regression results*	Catastrophic effects (1 = yes; 0 = no) Instrumented = diabetes mellitus (1 = yes; 0 = no)		Catastrophic effects (1 = yes; 0 = no) Instrumented = cardiovascular diseases (1 = yes; 0 = no)		Catastrophic effects(1 = yes; no = 0) Instrumented cancer (1 = yes; 0 = no)	
	Coefficient	SE	Coefficient	SE	Coefficient	SE
Diabetes mellitus	0.1998*	0.0224				
Cardiovascular diseases			0.2080*	0.0243		
Cancer					0.1133	0.0791
Gender	0.011*	0.003	0.0156*	0.0036	0.0040	0.0034
Years of education	0.0006	0.004	0.0005	0.0004	-0.0004	0.0004
Age	0.0012*	0.0002	0.0001	0.0002	0.0016*	0.0002
Household size	0.00365*	0.0017	0.0026*	0.0017	0.00278	0.0017
Number of children (age < 18 years)	0.0012	0.0013	0.0008	0.0013	0.0027*	0.0012
Household expenditure percentiles	-0.0088*	0.0016	-0.0089*	0.0017	-0.0087*	0.0015
Austria	0.0089	0.011	0.0109	.01110	0.0086	0.0109
Belgium	0.0200**	0.0114	0.0227*	0.0114	0.0226*	0.0113
Czech Republic	0.3792*	0.0112	0.3859*	0.0111	0.39564*	0.0109
Denmark	0.0804*	0.0168	0.0747*	0.0169	0.0818*	0.0167
France	-0.0101	0.0111	-0.0067	0.0111	-0.0080	0.0110
Germany	0.0314	0.0611	0.0444	0.0612	0.0179	0.0608
Hungary	0.6574*	0.0119	0.6493*	0.0121	0.6733*	0.0118
Italy	0.0296*	0.0126	0.0406*	0.0127	0.0340*	0.0126
Poland	0.3777*	0.0448	0.3736*	0.0449	0.37966*	0.0443
Portugal	0.1373*	0.0123	0.1561*	0.0122	0.1514	0.0120
Slovenia	-0.0196	0.0116	-0.0192*	0.0116	-0.0157	0.0115
Spain	-0.0084	0.0123	-0.0087	0.0123	0.0040	0.0122
Sweden	-0.0137	0.0352	-0.0230	0.0354	-0.0174	0.0348
Switzerland	0.0241*	0.0120	0.0274**	0.0120	0.0129	0.0118
Constant	-0.0878*	0.0197	-0.0355*	.0209	-0.0832*	0.0203
*First stage regression results*	Diabetes mellitus (1 = yes; 0 = no)		Cardiovascular diseases (1 = yes; 0 = no)		Cancer (1 = yes; 0 = no)	
	Coefficient	SE	Coefficient	SE	Coefficient	SE
Alcohol consumption	-0.0423*	0.0040	-	-		
Asian origin			-0.8112**	0.4696		
Middle-east origin	0.3367*	0.1550	-	-		
Smoking	0.1291*	0.0208	0.1449*	0.0198	0.1290*	0.0247
Body mass index	-0.0583*	0.0019	0.0254*	0.0019		
Physical activity			-	-	-0.0572*	0.0070
High level of cholesterol			0.3900*	0.0204		
Gender	-0.1872*	0.0214	-0.2175*	0.0195	0.1039*	0.0249
Age	0.0188*	0.0010	0.0360*	0.0090	0.0138*	0.0012
Years of education	-0.0159*	0.00253	-0.0142*	0.0024	0.0106*	0.0028
Household size	-0.0371*	0.0105	-0.0121	0.0102	-0.0296*	0.0134
Number of children (age < 18 years)	0.0187*	0.0072	0.0334*	0.0069	-0.0136	0.0091
Household expenditure percentiles	0.0042	0.0092	-0.0039	0.0088	-0.0061	0.0109
Austria	0.0036	0.072	0.0352*	0.0648	-0.0436	0.0759
Belgium	0.1053	0.0741	0.0197	0.0673	0.0434	0.0776
Czech Republic	0.2848*	0.0712	0.1484*	0.0648	-0.1443*	0.0767
Denmark	-0.129	0.128	0.1296*	0.1081	-0.1879	0.1343
France	0.0626	0.0726	0.0720	0.0657	-0.0825	0.0767
Germany	0.0864	0.3814	0.3253	0.3965	0.5463**	0.3205
Hungary	0.2445*	0.0749	0.4045*	0.0676	-0.2078*	0.0828
Italy	0.1011	0.0807	0.1909*	0.0573	-0.1813*	0.0907
Poland	0.0374	0.2780	0.1926	0.2447	-0.1301	0.3316
Portugal	0.3508*	0.0760	0.1432*	0.0714	-0.0936	0.0854
Slovenia	0.0228	0.0749	0.0597*	0.0675	-0.1120	0.0806
Sweden	0.0006	0.2183	0.2028	0.1811	0.0385	0.2348
Switzerland	-0.232*	0.0798	0.2832*	0.0717	0.0651	0.0815
Constant	-3.6308*	0.1405	-3.913*	0.1324	-2.531*	.1499

Statistically significant,

* p<0.01,

** p<0.05

Catastrophic health expenditures refer to the case when out-of-pocket payments exceed a certain threshold share of either total or non-food expenditure of households.

**Table 3:** Ordinary least square regression–“naïve model”.

	Catastrophic effects of health care costs					
	B	SE	B	SE	B	SE
Diagnosed diabetes within the eligible respondents	0.037*	0.004				
Diagnosed cardio-vascular disease within the eligible respondents			0.043*	0.020		
Diagnosed cancer within the eligible respondents					0.003*	0.006
Gender	0.004**	0.002	0.006**	0.002	0.003	0.002
Age	0.001*	0.000	0.001*	0.000	0.001*	0.000
Years of education	-0.001*	0.000	-0.001**	0.000	-0.001*	0.000
Household size	-0.003*	0.001	-0.003**	0.001	-0.003**	0.001
Number of children (age < 18 years)	0.004*	0.001	0.003*	0.001	0.004*	0.001
Household expenditure percentiles	-0.007	0.001	-0.007*	0.001	-0.007	0.001
Austria	0.012**	0.007	0.001	0.007	0.011	0.007
Belgium	0.025*	0.006	0.022*	0.006	0.025	0.006
Czech Republic	0.428*	0.006	0.428*	0.006	0.431	0.006
Denmark	0.106*	0.008	0.106*	0.008	0.105	0.008
France	0.000	0.006	-0.001	0.006	0.000	0.106
Germany	0.004	0.009	0.006	0.009	0.006	0.009
Hungary	0.678*	0.008	0.673*	0.008	0.680	0.008
Italy	0.031*	0.007	0.033*	0.007	0.032	0.007
Poland	0.433*	0.009	0.431*	0.009	0.434	0.009
Portugal	0.155*	0.008	0.156*	0.008	0.157	0.008
Slovenia	-0.008	0.008	-0.019	0.007	-0.008	0.008
Spain	0.001	0.007	0.003	0.007	0.002	0.007
Sweden	0.007	0.008	0.006	0.008	0.008	0.008
Switzerland	0.023*	0.007	0.024*	0.007	0.021	0.007
Constant	-0.019**	0.012	-0.016	0.012	-0.015	0.012

Statistically significant,

* p<0.01,

** p<0.05

Catastrophic health expenditures refer to the case when out-of-pocket payments exceed a certain threshold share of either total or non-food expenditure of households.

**Table 4:** Second-stage regression of treatment-effect model per country representing only coefficients for instrumented variables per country.

	Outcome variable for all three instrumented variables: catastrophic health expenditure					
	Instrumented variable: Diabetes mellitus		Instrumented variable: Cardiovascular diseases		Instrumented variable: Cancer	
	B	SE	B	SE	B	SE
Austria	0.097*	0.027	0.123*	0.038	n.e.	n.e.
Belgium	0.288*	0.041	0.074	0.048	0.182	0.116
Czech Republic	-0.219	0.160	0.003	0.093	-0.288	0.359
Denmark	n.e.	n.e.	n.e.	n.e.	0.098	0.714
France	0.027	0.020	0.046*	0.020	-0.095	0.079
Germany	-0.455	0.426	0.101	0.158	n.e.	n.e.
Hungary	0.378*	0.083	0.505*	0.104	0.994*	0.417
Italy	0.138	0.091	0.114	0.113	0.261**	0.150
Netherlands	0.137**	0.064	-0.084	0.055	0.024	0.168
Poland	0.384*	0.134	0.430	0.096	1.31*	0.504
Portugal	0.458*	0.112	0.653*	0.161	1.32*	0.596
Slovenia	-0.023	0.03	0.040	-0.041	0.029	0.016
Spain	0.064	0.049	0.064	0.055	n.e.	n.e.
Sweden	0.044	0.042	-0.157	0.161	0.088	0.130
Switzerland	0.105*	0.046	0.026*	0.038	0.284*	0.114

n.e. = the model cannot be estimated, no instruments passed the validity test;

* p<0.01,

** p<0.05

Catastrophic health expenditures refer to the case when out-of-pocket payments exceed a certain threshold share of either total or non-food expenditure of households.

**Table 5:** Correlations between the predicted probability of having chronic diseases and experiencing catastrophic health expenditure.

	Total health expenditure as a percentage of GDP	Out-of-pocket payments as a percentage of total health expenditure	Public health expenditure as a percentage of total health expenditure	Private health expenditure as a percentage of total health expenditure	Formal co-payments vs. informal co-payments
P(Expected catastrophic expenditure ǀ diabetes = 1)	-0.447	0.279	-0.231	-0.574*	0.398
Probability of diabetes = 1	-0.462	0.367	-0.277	-0.629*	0.332
P(Expected catastrophic expenditure ǀ cancer = 1)	-0.370	0.314	-0.293	-0.451	0.347
Probability of cancer = 1	0.565*	-0.260	0.318	0.311	-0.109
P(Expected catastrophic expenditure ǀ cardio = 1)	-0.324	0.330	-0.286	-0.389	0.406
Probability of cardio = 1	-0.337	0.281	-0.318	-0.604*	0.128
P(Expected catastrophic expenditure ǀ chronic_all = 1)	-0.309	0.315	-0.264	-0.393	0.423
Probability of chronic_all = 1	-0.170	0.177	-0.161	-0.510	0.166

* p<0.01

Catastrophic health expenditures refer to the case when out-of-pocket payments exceed a certain threshold share of either total or non-food expenditure of households.

The predicted probability indicating the probability of catastrophic health expenditure conditional on one of the three chronic diseases is present.

Table 2 presents the results for the treatment–effect models, including all countries for each of the three chronic diseases. The first model is related to diabetes mellitus. The results from the first stage regression show that being diagnosed with diabetes mellitus was associated with older people originating from the Middle East and those who smoke. On the other hand, older people who were diagnosed with diabetes mellitus consumed less alcohol than their counterparts who were not diagnosed with diabetes mellitus. A higher body-mass index was also negatively associated with diabetes mellitus. The results from the second-stage regression show that diabetes mellitus was significantly associated with catastrophic health expenditure. In comparison with the Netherlands, older people from Portugal, Poland, Denmark, Italy, Switzerland, Belgium, the Czech Republic and Hungary had a higher probability to experience the catastrophic effects of out-of-pocket payments.

The second model is related to cardiovascular diseases. The results from the first-stage regression show that having cardiovascular diseases is connected with older people who are smokers, who have a high level of cholesterol and those with a higher body mass index. Older people originating from Asia are less likely to be diagnosed with cardiovascular diseases. The results from the second-stage regression show that diagnosed cardiovascular diseases were associated with catastrophic health expenditure. Older people with diagnosed cardiovascular diseases from Belgium, Hungary, Chez Republic, Portugal, Slovenia, Denmark, Italy and Switzerland had a higher probability to experience catastrophic health expenditure than their counterparts in the Netherlands.

The third model is related to cancer. The results from the first-stage regression show that diagnosed cancer is associated with older people who smoke and negatively associated with people who report regular physical activity. The results from the second-stage regression show that diagnosed cancer was not significantly associated with catastrophic health expenditure.

Table 3 shows the results from the OLS regression. All three chronic diseases are significant predictors of the catastrophic health care expenditure in the OLS regression.

Table 4 presents only the coefficients for instrumented variables from the second-stage regression in the treatment-effect model per country. Diabetes mellitus is a significant predictor of catastrophic health expenditure in Austria, Belgium, Hungary, the Netherlands, Poland, Portugal and Switzerland. When all countries are compared, using the Netherlands as the base category, the country indicator for Austria is not significant. Diagnosed cardiovascular diseases are significant predictors for catastrophic health expenditure in Austria, France, Hungary, Portugal and Switzerland. When all countries are compared, using the Netherlands as the base category, the country indicators for France and Spain are not significant, while the country indicator for Slovenia is significant. Diagnosed cancer is a significant predictor for catastrophic health expenditure in Hungary, Italy, Poland, Portugal and Switzerland.

In Tables 2 and 4, our outcome variable is the indicator that was coded as 1 if the total amount of out-of-pocket payments per person per year exceeded 10% of total annual income per person. Since the threshold for assessing catastrophic health expenditure is arbitrary, we also present results on how the incidence of catastrophic health expenditure evolves in different countries when the thresholds vary from 5% to more than 40%. These results are presented in Fig 1. For the majority of the countries, when the threshold is higher than 15%, the incidence of catastrophic health expenditure is close to 0. However, in some countries, such as Portugal, the Czech Republic or Denmark, some parts of the population experience catastrophic health expenditure even when the threshold is set at 40% or more.

Table 5 presents the correlations between the predicted probability of having chronic diseases, experiencing catastrophic health expenditure, and macro indicators (presented in Table 1). We calculated the predicted probability as the probability of catastrophic health expenditures when one of three chronic diseases was diagnosed based on the SHARE dataset. The results show that a high share of public expenditure on health is negatively associated with the probability of having diabetes mellitus and experiencing catastrophic health expenditure. Similar results were observed for cardiovascular diseases.

## Discussion and Conclusions

The results from the treatment-effect model indicate that diagnosed diabetes mellitus and cardiovascular diseases are significant predictors for catastrophic health expenditure among older people in the 15 European countries included in this study. However, diagnosed cancer was not found to be a significant predictor for catastrophic health expenditure. One possible explanation is that the incidence of diagnosed diabetes mellitus and cardiovascular diseases among our sample is higher than that of diagnosed cancer. A second possible explanation is that patients with diagnosed cancer are usually exempted from official co-payments for interventions or therapies with confirmed benefits [46]. Finally, premature death is more common among cancer patients than among patients with other chronic diseases [46, 47]. However, it is worth noting that when the “naïve” model with OLS regression is applied, diagnosed cancer is a significant predictor of catastrophic health expenditure. Furthermore, the absolute coefficients of the disease-specific indicators for diabetes mellitus and cardiovascular diseases in the OLS regression are lower in comparison with the treatment-effect models. We believe that this difference in results justifies the use of the treatment-effect model.

Older people diagnosed with diabetes mellitus in Portugal, Poland, Denmark, Italy, Switzerland, Belgium, the Czech Republic and Hungary have a higher probability to experience catastrophic health expenditure than their counterparts in the Netherlands, which confirmed our expectations. Based on our assumptions outlined in the introduction section, a possible explanation is that the share of out-of-pocket payments as a percentage of total health expenditure is the lowest (6%) in the Netherlands in comparison with other countries, especially Portugal (32%), Switzerland (28%) and Hungary (27%). Moreover, co-payments in Portugal and Switzerland are obligatory for basic services, while in Hungary informal payments are widespread and government expenditure accounts for only 5% of GDP, compared to 10% in the Netherlands. A similar situation is observed in Poland, where co-payments are not obligatory, but the share of out-of-pocket payments as a percentage of total health expenditure is high (23%) and government expenditure as a percentage of GDP is a low 5%.

The results are similar with regard to diagnosed cardiovascular diseases. In comparison with the Netherlands, older people in Portugal, Switzerland, Denmark, Hungary, Italy, and Slovenia show a higher probability to experience catastrophic health expenditure.

Denmark presents an interesting case. Older people with diagnosed diabetes mellitus and cardiovascular diseases living in Denmark have a higher probability of catastrophic health expenditure than their counterparts in the Netherlands. However, when we estimated catastrophic health expenditure for people diagnosed with diabetes mellitus or cardiovascular diseases only for Denmark, the model was not significant. This could be explained by the fact that only small shares of older people in Denmark experience a financial burden resulting from chronic diseases. Outpatient and inpatient services in Denmark are generally provided free of charge and patients with chronic disease will have access to free medication, when their annual expenditure exceeds DKK 3600 (EUR 485) [48]. In the Netherlands, compulsory deductibles per year were EUR 350 in 2013, which means that patients could apply for reimbursement of drugs that are included in the Medical Reimbursement System after their annual expenditure crosses this ceiling [48]. Moreover, vulnerable groups, such as those with chronic illnesses, are partly compensated in the Netherlands for their co-payments [18].

The application of the treatment-effect model per country yielded some additional insights regarding catastrophic health expenditure among older people with chronic conditions in Europe. Diagnosed diabetes mellitus is a significant predictor for catastrophic health expenditure in Austria and the Netherlands, while diagnosed cardiovascular diseases is a significant predictor for catastrophic health expenditure in France and Spain. These countries are characterised by the absence of obligatory co-payments (Spain) or their low level (France, the Netherlands and Austria). They also have exemption mechanisms in place for older people and/or those with chronic illnesses. However, in some countries, like in France [49], the income-threshold for being eligible for full insurance coverage (without any co-payments) is very low. In this way, many people who are not exempted from co-payments because their income, although low, is still above the threshold, can experience catastrophic health expenditure. Furthermore, our results suggest that macro-indicators might be necessary, but not sufficient, mechanisms for protecting vulnerable groups such as older people from catastrophic health expenditure. Not only the magnitude and intensity but also the time when co-payments occur can play a role in experiencing catastrophic health expenditure [6]. If all co-payments need to be paid during a short period of time, the catastrophic effects can be greater. Even in wealthy countries with well-developed risk-pooling mechanisms and low levels of co-payments catastrophic health expenditure can be observed. Findings from [50] suggest that catastrophic health care expenditures occur in middle-income countries, countries in transition and countries with a high level of out-of-pocket patient payments. Based on the findings from this study, most of the previous research has focused on catastrophic health care expenditure and the role of chronic diseases and ageing in countries like Russia, Iran, Ukraine, Turkey and Mexico [15, 29, 32, 51, 52]. Among the “new” EU member states, attention has been paid to Estonia, Hungary and Poland [13, 53]. However, recent results have shown that older adults can face a financial burden even in highly developed countries such as Switzerland or the United States [54]. Although the study by Osbor et al., 2014, does not account for joint causality and does not report a catastrophic headcount ratio, it is line with our results. Findings from our study also suggest that the organization of the health system and of social protection measures are important for the financial burden, but they are sometimes not sufficient. For example, almost all countries in our sample that require official co-payments have some exemption mechanisms; yet, our results illustrate that these mechanisms are not always sufficient to protect individuals from catastrophic health expenditure.

Similar to previous studies [55, 56], our results show that older old people and those from lower income percentiles have a higher probability to experience catastrophic health expenditure. Recent studies have emphasized the possible negative effects of the economic crisis on the growing population of older people in European countries [54, 57]. Our results show that older people experience a financial burden due to health care consumption, including catastrophic health expenditure. It will be essential to better understand the specific reasons for catastrophic health expenditure in each of the countries examined, in order to protect older people with chronic conditions from this financial burden in the future.

The limitations of this study result from the way in which chronic diseases were categorised. For example, in case of diabetes mellitus, increased blood sugar level is not necessary identical with having the diagnosed disease. Furthermore, it was not possible to separate type I from type II diabetes mellitus. Another limitation of the study is that sample sizes of people diagnosed with diverse chronic diseases differ vastly across countries. Furthermore, we did not have information on whether out-of-pocket payments were specifically related to the diagnosed chronic diseases. Also, within the SHARE data it was not possible to identify which of out-of-pocket payments are spent on which drugs or which particular services are paid for inpatient care. Another limitation is related to the re-call period for out-of-pocket patient payments. Data for out-of-pocket patient payments are collected for a re-call period of 12 months and this may lead to an underestimation of total costs. Also, one of the limitations in this study is related to the use of imputation data. It is possible that imputing the data for a missing aggregate variable such as income can lead to bias [24]. In view of this, we have performed a sensitivity analysis using only non-imputed data. The results from the sensitivity analysis are compatible with the original analysis and they are presented in the supplementary materials (see Table E in S1 file and Table F in S1 file). Finally, we assessed catastrophic health expenditure using a threshold of 10% of total household income per person and year, since this is the most commonly used threshold in EU countries. As indicated in Fig 1 (where we varied the threshold), in most EU countries, out-of-pocket payments did not reach 5% of total household income per person. It also needs to be recognized that catastrophic health expenditure only occurs when people use health services. For those who needed care but did not use care due to financial or non-financial barriers, it was not possible to estimate the risk of catastrophic health expenditure.

## Supporting Information

S1 File Table A: Descriptive statistics for the total sample (N = 51661). Table B: Ordinary least square regression (OLS) for three instrumented variables and possible instruments. Table C: Validity test and overestimation test presented per different models. Table D: Out-of-pocket payments for different types of services and different chronic diseases. Table E: Results of the treatment-effect model (two-steps); dependent variable: catastrophic effects of health expenditure without imputed data. Table F: Results of the treatment-effects model (two-steps); dependent variable: catastrophic effects of health care expenditure without imputed data (continued).
(PDF)
