# Supplementary material for: Catastrophic Health Care Expenditure among Older People with Chronic Diseases in 15 European Countries
Source: PLoS One. 2016 Jul 5;11(7):e0157765. doi: 10.1371/journal.pone.0157765 (PMC4933384; doi:10.1371/journal.pone.0157765)
Supplement: S1 File — Table A: Descriptive statistics for the total sample (N = 51661). Table B: Ordinary least square regression (OLS) for three instrumented variables and possible instruments. Table C: Validity test and overestimation test presented per different models. Table D: Out-of-pocket payments for different types of services and different chronic diseases. Table E: Results of the treatment-effect model (two-steps); dependent variable: catastrophic effects of health expenditure without imputed data. Table F: Results of the treatment-effects model (two-steps); dependent variable: catastrophic effects of health care expenditure without imputed data (continued). (PDF) [file pone.0157765.s001.pdf]

Table A: Descriptive statistics for the total sample (N=51661)

	Frequencies	mean	minimum	minimum
Outcome variable				
Catastrophic effects of health care expenditure	1=catastrophic effect -yes (13.7%)<br>0=catastrophic effect-no (86.3%)	-	-	-
Instrumented variables				
Diagnosed diabetes within the eligible respondents	1=yes (12.5%) 0=no (87.5%)	-	-	
Diagnosed cardiovascular disease within the eligible respondents	1=yes (53.2%) 0=no (46.8%)	-	-	
Diagnosed cancer within the eligible respondents	1=yes (5.1%) 0=no (94.9%)	-	-	
Instrumental variables				
Asian origin	1=yes (0.1%); 0=no (99.9%)	-	-	-
Middle-east origin	1=yes (0.2%); 0=no (99.8%)	-	-	-
Ever smoked	1=yes (45.1%); 0=no (54.9%)	-	-	-
Alcohol consumption	From 0=not at all to 6=every day	-	-	-
Body mass index	Continues	26.3	14.79	88.38
Physical activity	From 0=never to 3=more than once per week	-	-	-
Covariates				
Household size	From 1 up to 12	-	-	
Gender	1=male (43.8%); 0=female (56.2%)	-	-	
Age	From 30 up to 106	67.00	15	98
Children	From 0 up to 17	-	-	-
Expenditure percentiles	Percentiles (1-5)*	-	-	-
Years of education	From 0 to 25	-	-	-
Country indicator Austria (N=5286)	1=yes (10.2%); 0=no (89.8%)	-	-	-
Country indicator Belgium(N=5300)	1=yes (10.3%); 0=no (89.7%)	--	-	-
Country indicator Czech Republic (N= 6118)	1=yes (11.8%); 0=no (88.2%)	-	-	-
Country indicator Denmark(N=2276)	1=yes (4.4%); 0=no (95.6%)	-	-	-
Country indicator Hungary(N=3076)	1=yes (6.0%); 0=no (94.0%)	-	-	-
Country indicator Italy(N=3583)	1=yes (0.1%); 0=no (99.9%)	-	-	-
Country indicator France(N=5857)	1=yes (11.3%); 0=no (88.7%)	-	-	-
Country indicator Germany(N=1572)	1=yes (3.0%); 0=no (97.0%)	-	-	-
Country indicator Netherlands(N=2762)	1=yes (5.3%); 0=no (94.7%)	-	-	-
Country indicator Poland(N=1724)	1=yes (3.3%); 0=no (96.7%)	-	-	-
Country indicator Portugal(N=2080)	1=yes (4.0%); 0=no (96.0%)	-	-	-
Country indicator Sweden(N=1951)	1=yes (3.8%); 0=no (96.2%)	-	-	-
Country indicator Spain(N=3570)	1=yes (6.9%); 0=no (93.1%)	-	-	-
Country indicator Slovenia (N=2756)	1=yes (5.3%); 0=no (94.7%)	-	-	-
Country indicator Switzerland(N=3750)	1=yes (7.3%); 0=no (92.7%)	-	-	-

Percentiles are based on the total sample

Table B: Ordinary least square regression (OLS) for three instrumented variables and possible instruments

	Diagnosed diabetes within the households		Diagnosed cardio-vascular disease within the households		Diagnosed cancer within the household	
	B	SE	B	SE	B	SE
Asian origin	-0.038	0.04	-0.125*	0.004	-0.006	0.032
Middle-east origin	0.031*	0.02	-0.020	0.035	-0.018	0.023
Ever smoked	0.019*	0.04	0.028*	0.004	0.009*	0.003
Alcohol consumption	-0.007*	0.001	-0.001	0.001	0.000	0.001
Body mass index	0.013*	0.01	0.005*	0.001	0.000	0.000
Physical activity	-0.017	0.01	-0.035	0.02	-0.009*	0.01
Cholesterol	0.108	0.004	0.095*	0.05	0.013	0.003

*p<0.05

B = unstandardized B coefficient = average change in dependent variable for one unit change in independent variable

SE = standard error of estimates

Table C: Validity test and overestimation test presented per different models

	Validity (Hausman) test	Overestimation test (Sargan score)
Model 1 (N=51661)<br>Outcome variable: Catastrophic effects of health care costs<br>Instrumented variable: Diagnosed diabetes within the households	chi2(1) = 164.77<br>Prob > chi2 = 0.0000	chi2(3) = 9.07 (p = 0.03)
Model 2 (N=51661)<br>Outcome variable: Catastrophic effects of health care costs<br>Instrumented variable: Diagnosed cardiovascular disease within the households	chi2(1) = 76.45<br>Prob > chi2 = 0.0000	chi2(2) = 16. 0552(p = 0.01)
Model 3 (N=51661) Outcome variable: Catastrophic effects of health care costs<br>Instrumented variable: Diagnosed cancer within the household	chi2(1) = 34.08<br>Prob > chi2 = 0.0000	chi2(1) = 1.30 (p = 0.10)

Table D: Out-of-pocket payments for different types of services and different chronic diseases (all amounts are expressed in Euros)

Total sample (N=51661)	Payments for inpatient care	Payments for outpatient care	Payments for prescribed drugs	Payments for nursing homes	Payments for home care services
	Mean(SD)	Mean(SD)	Mean(SD)	Mean(SD)	Mean(SD
Diagnosed diabetes mellitus	21.2(324.35)	40.76(305.21)	150.74(402.82)	13.10 (349.90)	22.73 (375.10
Diagnosed high blood pressure	29.13(404.25)	42.74(327.53)	120.19(294.26)	10.38(353.89)	10.94(260.55)
Diagnosed high blood cholesterol	29.08(381.68)	55.76(625.97)	128.67(333.87)	9.08(299.67)	10.88(294.26)
Diagnosed heart attack	37.18(397.78)	47.64(291.07)	149.29(386.58)	29.39(781.75)	24.14(428.27)
Diagnosed stroke	53.59(650.65)	45.29(245.65)	173.24(319.60)	81.14(1176.91)	89.97(897.90)
Diagnosed cardiovascular diseases (all types together)	30.48(392.76)	48.89(473.60)	116.94(283.38)	13.07(456.51)	15.36(366.72)
Diagnosed cancer	73.81(582.47)	95.77(1123.80)	148.68(434.52)	15.60(568.15)	32.64(588.10)
**Country Austria**					
Diagnosed diabetes mellitus	82.39(207,99)	122.00(784.97)	296.53(286.65)	47.81(315.53)	446.72(1916,71)
Diagnosed high blood pressure	106.96(398.47)	126.64(440.09)	225.49(260.68)	66.86(1189.89)	149.48(1274.46)
Diagnosed high blood cholesterol	100.54(407.40)	163.04(563.02)	235.31(317.83)	20.24(157.39)	197.25(1634.52)
Diagnosed heart attack	213.14(894.73)	243.30(933.18)	296.76(296.49)	221.57(2104.87)	484.94(2279.83)
Diagnosed stroke	132.73(218.64)	200.59(720.29)	352,53(341.62)	435.32(2997.14)	1105.74(3546.44)
Diagnosed cardiovascular diseases (all types together)	118.25(538.44)	158.01(616.88)	225.86(280.28)	60.33(1016.59)	290.83(1939.19)
Diagnosed cancer	259.44(1192.22)	238.01(604.29)	283.30(400.38)	336.56(2924.76)	578.39(2825.09)
**Country Belgium**					
Diagnosed diabetes mellitus	80.95(253.06)	66.43(391.14)	282.71(556.23)	49.61(957.72)	25.34(158.76)
Diagnosed high blood pressure	99.46(949.86)	40.38(244.51)	207.48(412.08)	23.08(509.39)	12.12(102.31)
Diagnosed high blood cholesterol	87.87(744.63)	41.35(180.81)	188.12(398.98)	26.56(552.75)	11.46(101.77)
Diagnosed heart attack	102.21(391.07)	43.23(187.72)	265.45(474.63)	63.16(1047.99)	22.41(136.59)
Diagnosed stroke	67.98(256.74)	56.54(232.64)	285,65(502.48)	427.45(2835.62)	31.47(196.24)
Diagnosed cardiovascular diseases (all types together)	91.31(760.82)	42.75(229.74)	193.35(409.32)	36.72(747.35)	12.09(106.00)
Diagnosed cancer	141.13(409.95)	51.48(167.43)	219.21(457.39)	12.48(112.95)	21.98(159.50)

	Payments for inpatient care	Payments for outpatient care	Payments for prescribed drugs	Payments for nursing homes	Payments for home care services
**Country Czech Republic**	Mean(SD)	Mean(SD)	Mean(SD)	Mean(SD)	Mean(SD)
Diagnosed diabetes mellitus	12.43(50.19)	14.37(79.42)	78.42(121.4)	4.62 (59.59)	12.92 (205.12)
Diagnosed high blood pressure	7.81(33.45)	10.61(49.88)	62.43(95.89)	2.38(37.97)	6.15(127.89)
Diagnosed high blood cholesterol	8.04(38.39)	9.20(24.52)	62.81(92.21)	1.54(16.37)	8.34(133.84)
Diagnosed heart attack	13.81(50.20)	12.01(53.82)	80.19(109.22)	4.86(62.99)	13.08(212.74)
Diagnosed stroke	19.91(72.19)	9.22(21.85)	80.11(105.89)	11.05(102.86)	50.62(428.94)
Diagnosed cardiovascular diseases (all types together)	7.86(33.94)	10.96(52.81)	60.01(93.79)	2.24(35.12)	6.76(134.41)
Diagnosed cancer	16.51(43.74)	13.25(28.34)	86.83(146.75)	0.62(9.7)	16.04(147.51)
**Country Denmark**					
Diagnosed diabetes mellitus	0.00(0.00)	7.85(58.59)	226.18(471.44)	63.16(733.96)	2.53(31.42)
Diagnosed high blood pressure	0.00(0.00)	15.16(150.07)	173.85(346.05)	28.87(603.31)	0.82(16.36)
Diagnosed high blood cholesterol	0.00(0.00)	20.15(182.64)	189.29(382.05)	0.41(6.73)	0.05(1.22)
Diagnosed heart attack	0.00(0.00)	20,88(130.78)	204,51(325.00)	118,88(1319.99)	0.00(0.00)
Diagnosed stroke	319.67(2496.72)	0.00(0.00)	271.62(467.69)	149.18(1165.14)	0.00(0.00)
Diagnosed cardiovascular diseases (all types together)	21.03(640.46)	13.15(133.14)	167.92(329.87)	31.48(600.24)	0.64(14.15)
Diagnosed cancer	325.00(2517.44)	6.93(38.85)	221.80(448.38)	4.55(35.24)	0.00(0.00)
**Country Hungary**					
Diagnosed diabetes mellitus	6.18(35.18)	5.99(77.99)	188.28(270.98)	0.15(2.06)	1.30(14.81)
Diagnosed high blood pressure	6.32(67.48)	10.44(256.08)	142.04(208.57)	0.76(12.51)	2.20(51.49)
Diagnosed high blood cholesterol	7.61(77.42)	3.33(20.71)	147.32(202.21)	0.26(6.64)	4.02(80.22)
Diagnosed heart attack	11.74(103.80)	4.43(36.32)	197.57(248.73)	0.36(6.82)	4.81(80.61)
Diagnosed stroke	12.77(124.36)	6.78(49.98)	208.65(251.49)	1.05(11.56)	1.38(14.25)
Diagnosed cardiovascular diseases (all types together)	7.40(74.41)	9.71(234.54)	140.82(208.05)	0.67(11.47)	1.95(47.11)
Diagnosed cancer	12.96(49.49)	11.39(90.02)	206.44(289.84)	0.00(0.00)	0.70(8.3)

	Payments for inpatient care	Payments for outpatient care	Payments for prescribed drugs	Payments for nursing homes	Payments for home care services
	Mean(SD)	Mean(SD)	Mean(SD)	Mean(SD)	Mean(SD)
**Country Italy**					
Diagnosed diabetes mellitus	3.82(73.51)	47.59 (206.56)	184.34 (345.59)	18.77(343.21)	33.97(497.91)
Diagnosed high blood pressure	16.38(390.91)	72.08(648.86)	159.96(331.31)	5.71(193.08)	4.05(48.98)
Diagnosed high blood cholesterol	5.66(114.86)	89.40(838.01)	166.28(307.97)	0.98(16.68)	2.02(32.17)
Diagnosed heart attack	1.60(23.32)	59.17(209.56)	191.27(270.23)	24.35(396.73)	5.12(45.77)
Diagnosed stroke	18.07(160.64)	78.31(217.68)	255.12(354.84)	3.61(32.92)	60.24(283.67)
Diagnosed cardiovascular diseases (all types together)	13.30(342.46)	68.78(578.06)	158.70(315.35)	4.90(168.75)	5.61(75.13)
Diagnosed cancer	18.92(149.86)	75.22(260.89)	257.41(469.44)	6.93(47.82)	18.46(114.20)
**Country France**					
Diagnosed diabetes mellitus	15.48(117.40)	78.26(513.38)	27.62(101.14)	0.39(5.25)	0.52(7.96)
Diagnosed high blood pressure	14.14(130.95)	93.02(589.11)	28.21(91.93)	11.09(355.20)	3.43(63.89)
Diagnosed high blood cholesterol	11.00(86.77)	98.14(509.58)	30.48(97.64)	12.60(388.72)	2.93(65.63)
Diagnosed heart attack	12.18(93.91)	69.90(319.98)	30.09(112.71)	4.10(97.42)	4.31(45.83)
Diagnosed stroke	6.70(60.09)	30.61(102.99)	34.55(116.95)	0.52(4.33)	4.11(42.14)
Diagnosed cardiovascular diseases (all types together)	11.38(110.05)	85.60(528.41)	28.13(91.67)	7.78(28.14)	2.51(51.44)
Diagnosed cancer	32.38(254.20)	91.28(472.67)	38.07(115.71)	0.12(1.00)	2.82(42.62)
**Country Germany**					
Diagnosed diabetes mellitus	35.92(132.29)	55.39(294.61)	111.39(229.56)	6.11(65.22)	14,38(131.41)
Diagnosed high blood pressure	20.27(63.33)	47.01(216.39)	91.24(190.84)	2.17(37.30)	2.75(28.37)
Diagnosed high blood cholesterol	20.73(67.46)	66.68(274.67)	90.94(128.22)	0.48(8.53)	2.13(25.04)
Diagnosed heart attack	40.28(139.52)	63.31(254.15)	108.65(249.89)	2.84(34.03)	19.12(169.82)
Diagnosed stroke	41.30(171.93)	128.96(478.14)	138.76(324.46)	32.65(235.47)	60.11(284.94)
Diagnosed cardiovascular diseases (all types together)	22.39(82.11)	51.28(222.51)	93.23(196.38)	3.78(67.88)	4.99(74.68)
Diagnosed cancer	55.82(157.83)	75.21(296.52)	122.05(309.59)	2.28(16.77)	25.54(191.94)

	Payments for inpatient care	Payments for outpatient care	Payments for prescribed drugs	Payments for nursing homes	Payments for home care services
	Mean(SD)	Mean(SD)	Mean(SD)	Mean(SD)	Mean(SD)
**Country Netherlands**					
Diagnosed diabetes mellitus	9.99(80.68)	33.79(207.61)	66.12(97.06)	37.78(613.14)	14.47(73.84)
Diagnosed high blood pressure	5.28(36.46)	29.93(153.04)	67,79(98,32)	3.63(48.24)	26.70(205.53)
Diagnosed high blood cholesterol	5.02(37.64)	146.68(2343.46)	65.78(93.57)	0.13(2.79)	18.17(210.31)
Diagnosed heart attack	7.96(42.70)	27.79(174.38)	74.78(109.27)	14.02(192.72)	25.94(160.59)
Diagnosed stroke	23.58(125.42)	89.70(317.61)	57.20(97.53)	3.50(27.04)	93.21(481.08)
Diagnosed cardiovascular diseases (all types together)	6.32(49.16)	84.63(1523.79)	68.08(99.65)	5.36(102.12)	23.98(186.36)
Diagnosed cancer	6.98(33.82)	447.19(4546.44)	63.78(88.28)	0.00(0.00)	5.47(28.04)
**Country Poland**					
Diagnosed diabetes mellitus	0.00(0.00)	1.21(8.84)	263.51(267.42)	0.00(0.00)	0.23(3.33)
Diagnosed high blood pressure	0.22(3.51)	2.21(18.01)	236.05(245.52)	0.05(1.41)	0.07(1.87)
Diagnosed high blood cholesterol	1.18(19.57)	1.30(12.71)	243.34(246.64)	0.10(1.94)	0.14(2.58)
Diagnosed heart attack	1.72(23.62)	1.88(15.64)	273.52(283.59)	0.00(0.00)	0.20(3.12)
Diagnosed stroke	0.00(0.00)	2.14(11.46)	304.02(276.92)	0.00(0.00)	0.00(0.00)
Diagnosed cardiovascular diseases (all types together)	0.58(12.67)	2.05(17.47)	224.30(240.36)	0.04(1.23)	0.05(1.64)
Diagnosed cancer	0.00(0.00)	6.12(42.00)	207.47(232.94)	0.00(0.00)	0.00(0.00)
**Country Portugal**					
Diagnosed diabetes mellitus	5.64(59.49)	6.4(64.53)	400.41(903.81)	8.67(74.24)	3.02(32.57)
Diagnosed high blood pressure	51.90(549.21)	6.46(54.33)	312.16(683.08)	18.91(439.77)	19.61(438.76)
Diagnosed high blood cholesterol	31.61(446.07)	5.21(49.86)	314.59(702.17)	8.68(116.65)	2.08(26.368)
Diagnosed heart attack	31.17(278.91)	11.33(82.91)	517.59(1154.74)	7.65(88.24)	3.45(36.55)
Diagnosed stroke	30.69(269.52)	5.05(24.97)	310.60(379.00)	3.48(32.34)	30.69(216.12)
Diagnosed cardiovascular diseases (all types together)	40.63(466.71)	6.74(57.10)	284.19(593.77)	16.59(374.36)	15.75(369.03)
Diagnosed cancer	35.09(298.38)	17.52(111.19)	502.80(1376.11)	0.00(0.00)	15.29(154.46)

	Payments for inpatient care	Payments for outpatient care	Payments for prescribed drugs	Payments for nursing homes	Payments for home care services
	Mean(SD)	Mean(SD)	Mean(SD)	Mean(SD)	Mean(SD)
**Country Sweden**					
Diagnosed diabetes mellitus	5.94(18.53)	36.63(40.52)	11.99(26.79)	0.57(7.32)	0.00(0.00)
Diagnosed high blood pressure	4.45(14.71)	39.55(40.49)	17.85(33.49)	0.19(4.02)	0.26(4.70)
Diagnosed high blood cholesterol	5.89(17.77)	40.00(40.44)	16.60(32.76)	0.08(1.35)	0.01(0.008)
Diagnosed heart attack	5.67(14.85)	40.15(42.39)	12.86(30.58)	0.06(0.57)	0.83(8.07)
Diagnosed stroke	9,09(22.94)	33,17(42.63)	14,12(33.06)	0.07(0.40)	1.52(12.81)
Diagnosed cardiovascular diseases (all types together)	4.62(15.06)	39.05(40.43)	16.63(32.61)	0.16(3.49)	0.27(4.65)
Diagnosed cancer	6.44(13.80)	49.03(49.60)	13.80(35.86)	0.03(0.20)	0.97(8.00)
**Country Spain**					
Diagnosed diabetes mellitus	1.72(41.1)	6.08(63.36)	36.23(135.26)	8.49(197.43)	7.64(94.48)
Diagnosed high blood pressure	2,64(83.29)	2,37(41.49)	33,63(121.56)	7,65(187.41)	4,47(70.57)
Diagnosed high blood cholesterol	1.9(37.42)	2.48(39.47)	43.05(135.40)	4.82(151.48)	6.21(87.85)
Diagnosed heart attack	0.01(0.24)	2.67(34.98)	29.29 (106.41)	15.59(249.17)	7.92 (89.12)
Diagnosed stroke	0.05(0.51)	0.05(0.04)	15.82(94.54)	17.57(172.60)	12.54(121.83)
Diagnosed cardiovascular diseases (all types together)	2.65(73.87)	2.92(45.50)	34.79(118.07)	6.16(160.17)	4.34(70.19)
Diagnosed cancer	0.10(0.88)	27.67(262.16)	84.47(523,34)	13.33(154.34)	4.53(51.34)
**Country Switzerland**					
Diagnosed diabetes mellitus	162.16(639.2)	217.41(661.51)	201.69(510.56)	3.58(36.00)	14.25(122.06)
Diagnosed high blood pressure	109.47(667,02)	152.08(428.28)	141.37(334.77)	22.33(514.98)	9.65(121.67)
Diagnosed high blood cholesterol	101.32(494.28)	206.92(711.25)	179.48(426.66)	37.42(694.73)	4.31(70.61)
Diagnosed heart attack	154.10(710.21)	237.63(649.79)	220.98(517.83)	188.78(2571.91)	18.07(137.03)
Diagnosed stroke	295.89(1808.52)	158.58(338.99)	249.28(562.23)	224.45(1794.85)	66.16(303.00)
Diagnosed cardiovascular diseases (all types together)	114.42(658.01)	174.55(561.15)	147.95(345.74)	45.03(1140.19)	9.03(114.51’
Diagnosed cancer	209.61(632.60)	233.78(467.51)	112.05(236.77)	0.75(11.07)	7.11(60.51)

Table E: Results of the treatment-effect model (two-steps); dependent variable: catastrophic effects of health expenditure (without imputed data)

Second-stage regression results	Catastrophic effects (1=yes; 0=no) Instrumented=diabetes mellitus (1=yes; 0=no)		Catastrophic effects (1=yes; 0=no) Instrumented=cardiovascular diseases (1=yes; 0=no)		Catastrophic effects(1=yes; no=0) Instrumented cancer (1=yes; 0=no)	
	Coefficient	SE	Coefficient	SE	Coefficient	SE
Diabetes mellitus	0.2651*	0.0243				
Cardiovascular diseases			0.3408*	0.0243		
Cancer					0.3206	0.0842
Gender	0.0347*	0.0034	0.0441*	0.0039	0.0237	0.0037
Years of education	0.0014*	0.0004	0.0014*	0.0005	-0.0001	0.0004
Age	0.0014*	0.0002	0.0004	0.0002	0.0016*	0.0002
Household size	-0.0286*	0.0019	0.0301*	0.0019	-0.0292*	0.0019
Number of children (age < 18 years)	0.0034**	0.0014	0.0023	0.0014	0.0057*	0.0014
Household expenditure percentiles	-0.0054*	0.0017	-0.0054*	0.0017	-0.0051*	0.0017
Austria	0.0609*	0.0121	0.0643*	0.1122	0.0613*	0.0120
Belgium	0.0453*	0.0124	0.0484*	0.0126	0.0466*	0.0124
Czech Republic	-0.0067	0.0122	0.0004*	0.0112	0.0171	0.0120
Denmark	-0.0068	0.0184	-0.0159*	0.0186	-0.0022	0.0184
France	-0.0439*	0.0121	-0.0389*	0.0123	-0.0405*	0.0121
Germany	-0.0873	0.0665	-0.0665	0.0676	-0.01225	0.0468
Hungary	0.1037*	0.0129	0.0862*	0.0133	0.1281*	0.0129
Italy	0.0591*	0.0138	0.0764*	0.0141	0.0678*	0.0138
Poland	-0.0170	0.0478	0.0238	0.0496	-0.1210	0.04872
Portugal	0.0664*	0.0134	0.0931*	0.0135	0.0865	0.0133
Slovenia	-0.0680	0.0126	-0.0682*	0.0128	-0.0604*	0.0127
Spain	-0.0561*	0.0134	-0.0314**	0.0136	0.0368	0.0134
Sweden	-0.0766	0.0384	-0.0907**	0.0354	-0.0814	0.1343
Switzerland	0.0585*	0.0181	0.0671*	0.0133	0.0421*	0.0130
Constant	-0.0264	0.0215	0.0604**	0.0220	-0.0079	0.0223

Statistically significant, $p<0.01$*, ** $p<0.05$*

Table F: Results of the treatment-effects model (two-steps); dependent variable: catastrophic effects of health care expenditure (continued)

First stage regression results	Diabetes mellitus (1=yes; 0=no)		Cardiovascular diseases (1=yes; 0=no)		Cancer (1=yes; 0=no)	
	Coefficient	SE	Coefficient	SE	Coefficient	SE
Alcohol consumption	-0.0423*	0.0040	-	-		
Asian origin			-0.8387**	0.4829		
Middle-east origin	0.3367*	0.1550	-	-		
Smoking	0.1291*	0.0208	0.2110*	0.0190	0.1290*	0.0247
Body mass index	0.0584*	0.0019	0.0211*	0.0019		
Physical activity			-	-	-0.0572*	0.0070
High level of cholesterol			0.3112*	0.0210		
Gender	-0.1872*	0.0214	-0.2175*	0.0195	0.1039*	0.0249
Age	0.0188*	0.0010	0.0354*	0.0090	0.0138*	0.0012
Years of education	-0.0159*	0.00253	-0.0104*	0.0024	0.0106*	0.0028
Household size	-0.0371*	0.0105	0.0123	0.0101	-0.0296*	0.0134
Number of children (age < 18 years)	0.0187*	0.0072	0.0315*	0.0069	-0.0136	0.0091
Household expenditure percentiles	0.0042	0.0092	-0.0003	0.0008	-0.0061	0.0109
Austria	0.0036	0.072	0.0042	0.0647	-0.0436	0.0759
Belgium	0.1053	0.0741	0.0248	0.0671	0.0434	0.0776
Czech Republic	0.2848*	0.0712	0.1014	0.0647	-0.1443*	0.0767
Denmark	-0.129	0.128	0.1284	0.1080	-0.1879	0.1343
France	0.0626	0.0726	-0.0690	0.0656	-0.0825	0.0767
Germany	0.0864	0.3814	-0.3355	0.3973	0.5463**	0.3205
Hungary	0.2445*	0.0749	0.3413*	0.0676	-0.2078*	0.0828
Italy	0.1011	0.0807	0.1964*	0.0759	-0.1813*	0.0907
Poland	0.0374	0.2780	0.1893	0.2446	-0.1301	0.3316
Portugal	0.3508*	0.0760	-0.1427**	0.0713	-0.0936	0.0854

First stage regression results	Diabetes mellitus (1=yes; 0=no)		Cardiovascular diseases (1=yes; 0=no)		Cancer (1=yes; 0=no)	
	Coefficient	SE	Coefficient	SE	Coefficient	SE
Slovenia	0.0228	0.0749	0.0374	0.0674	-0.1120	0.0806
Sweden	0.0006	0.2183	0.1819	0.1818	0.0385	0.2348
Switzerland	-0.232*	0.0798	0.2729*	0.0715	0.0651	0.0815
Constant	-3.6308*	0.1405	-4.270*	0.1251	-2.531*	.1499

Statistically significant, p<0.01, ** p<0.05*
